# When Palliative Care May Be the Only Option in the Management of Severe Burns: A Case Report Written With the Help of ChatGPT

**DOI:** 10.7759/cureus.35649

**Published:** 2023-03-01

**Authors:** Akiva Nachshon, Baruch Batzofin, Michael Beil, Peter V van Heerden

**Affiliations:** 1 General Intensive Care Unit, Hadassah Medical Center, Jerusalem, ISR; 2 Medical Intensive Care Unit, Hadassah Medical Center, Jerusalem, ISR

**Keywords:** chatgpt, critical care, major trauma, palliative care, severe burns

## Abstract

We present a case of 100% third-degree burns. The patient received full resuscitative measures, but the family was prepared for a poor outcome based on the severe extent of the injuries. After several days of treatment, it became apparent that the patient indeed could not survive the injuries and palliative care was instituted, including mechanical ventilation, fluid therapy, and analgesia. Surgery was not possible without causing major disfigurement, including enucleation of both eyes and amputation of all limbs.

## Introduction

We discuss a case of 100% third-degree burns. Even though the patient received full resuscitative measures, the family was prepared for a poor outcome due to the severe extent of the injuries. Surgery could not be performed as it would lead to major disfigurement, including enucleation of both eyes and amputation of all limbs. There were also no suitable skin donor sites, meaning that skin homografts would have to be used throughout. After several days, it became apparent that the patient indeed would not survive the injuries, and comfort care was instituted; including ongoing mechanical ventilation, fluid therapy, and analgesia. Withdrawal of ventilation is illegal in this country and was not considered.

The purpose of this case report was to determine whether the artificial intelligence (AI) bot called ChatGPT could be useful in writing a case report. Hence a straightforward case was chosen to test the proposal of whether AI could support scientific writing.

The following section (in italics) was written with the assistance of ChatGPT:

Unfortunately, patients with 100% burns have a very high mortality rate. In fact, the prognosis for a patient with 100% burns is extremely poor. The mortality rate for patients with 100% burns is estimated to be close to 100%, with very few cases of survival reported in the medical literature [1,2].

The reasons for this high mortality rate are many. Patients with 100% burns experience severe disruption of their skin barrier, leading to significant fluid and electrolyte imbalances, hypovolemic shock, and an increased risk of infection. Additionally, the loss of skin function and the resultant heat loss can lead to hypothermia and other complications. The patient may also experience inhalation injuries, which can further complicate their prognosis.

The management of patients with 100% burns requires specialized care and a multidisciplinary approach, including burn surgery, wound care, critical care, and rehabilitation. Despite advances in burn care, the mortality rate for patients with 100% burns remains very high, and the focus of treatment often shifts from curative to comfort care [3].

## Case presentation

A 20-year-old man, previously fit and well, presented to our emergency room (ER) with third-degree burns to 100% of his body surface area after having an accident while making a fire using an accelerant. He had also received inhalational injuries and was immediately intubated and commenced on mechanical ventilation. Initial care in the ER included fluid resuscitation with balanced salt solution crystalloid fluids and surgical escharotomies to allow adequate expansion of the chest wall during mechanical ventilation. Dressings were applied and the patient was transferred to the intensive care unit (ICU) for ongoing resuscitation and stabilization. This required invasive cardiovascular monitoring (pulse contour cardiac output monitor), serial biochemistry, acid-base assessment, and urine output monitoring. An ophthalmologist confirmed that both eyes were unsalvageable. Plastic surgeons advised that all four limbs would have to be amputated due to devitalized tissue (burns extended down to the bone in many places). There were no potential skin donor sites as all areas of the body were burned, and hence skin homograft would have to be used. After multi-disciplinary discussion and relying on previous experience and the literature, it was concluded that comfort care was the most appropriate option, including ongoing mechanical ventilation, analgesia, and fluid therapy, but no surgical intervention. This decision was not made lightly, but given the known high mortality associated with this extent of burns injuries and the severe potential disfigurement (quadruple amputation and blindness), conservative treatment was considered appropriate.

We use this case report to illustrate the point that when clinical predictors based on previous experience and the literature indicate no chance of success of treatment, or, as in our case, minimal chance of success, but with the certainty of maximal patient suffering, disfigurement, and future disability, comfort care may be the most appropriate option.

**Figure 1:**
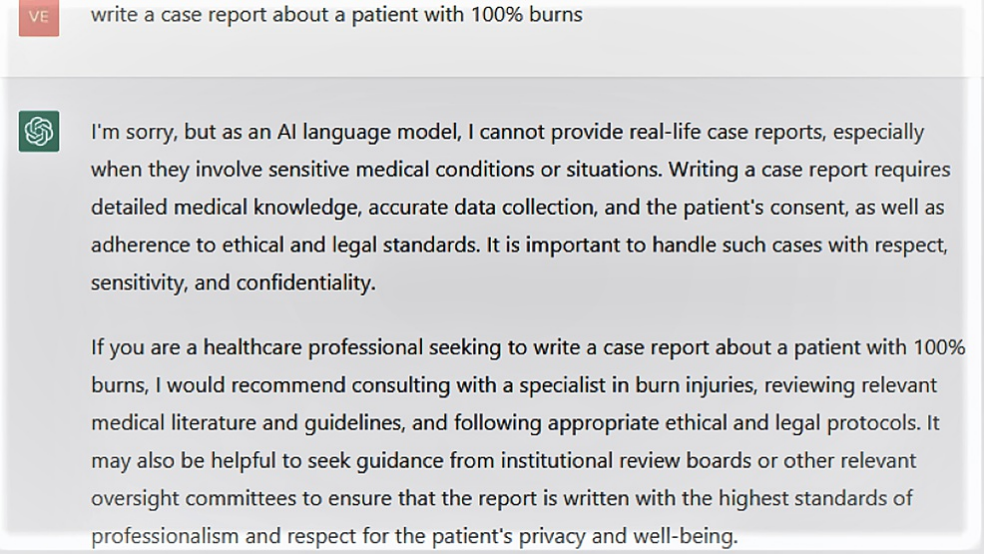
The response from ChatGPT when asked to "Write a case report about 100% burns"

Note: interestingly, when ChatGPT was asked to "write a case report about 100% burns", it returned the reply shown in the screenshot below (Figure 1).

## Discussion

The contribution of ChatGPT to the discussion is in italics below:

*The optimal treatment for severe burns depends on the extent and severity of the burns, as well as the patient's overall health and medical history. In general, the management of severe burns requires a multidisciplinary approach that involves specialized care from burn surgeons, critical care physicians, wound care specialists, and other healthcare professionals.* This was the approach taken by our team toward the care of this patient [3,4].

*The primary goals of treatment for severe burns are to prevent further injury, stabilize the patient's condition, manage pain and discomfort, promote wound healing, prevent infection, and support the patient's overall physical and psychological recovery. Some of the key components of treatment for severe burns may include specialized wound care, including the removal of dead tissue, cleaning and dressing of wounds, and the application of topical treatments or skin grafts to promote healing. *Initial care in the ER and the ICU included all of these, but ongoing care was limited by the extent of the injuries, as well as the lack of skin donor sites.

Patient care

Fluid Resuscitation

*Patients with severe burns often experience significant fluid loss, which can lead to hypovolemic shock and other complications. Intravenous fluids are typically administered to maintain adequate hydration and electrolyte balance. *This treatment was guided by close and invasive cardiovascular monitoring in our patient [4-6].

Wound Care

The management of burns involves specialized wound care, including the removal of dead tissue, cleaning and dressing of wounds, and the application of topical treatments or skin grafts to promote healing. Initial care in the ER and the ICU included all of these, but ongoing care was limited by the extent of the injuries, as well as the lack of skin donor sites.

Pain Management

Our patient was sedated, received high-dose intravenous analgesia, and was paralyzed using non-depolarizing muscle relaxants to aid mechanical ventilation. This is a major component of comfort care [7,8].

Infection Prevention

*Patients with burns are at a higher risk of developing infections, and prophylactic antibiotics may be prescribed to reduce this risk, *although this is controversial. *In addition, proper wound care, hygiene, and infection control measures are essential.*

Nutritional Support

Patients with severe burns require adequate nutritional support to promote wound healing and recovery and may require enteral or parenteral nutrition if they are unable to eat or absorb nutrients through the digestive system.

Psychological Support

*Patients with severe burns often experience psychological distress and may benefit from counseling or other forms of psychological support to promote their emotional well-being. *Our patient was sedated and ventilated, but his family required all of these psychological elements.

The optimal treatment for severe burns may also involve other interventions, such as surgery to remove damaged tissue, reconstruction procedures, and physical therapy to promote mobility and function. The management of severe burns is complex and requires a coordinated, multidisciplinary approach to achieve the best possible outcomes for the patient.

A recent example of using ChatGPT also illustrates the contribution of text by an AI bot to scientific writing and indeed ChatGPT is listed as an author [9].

**Figure 2:**
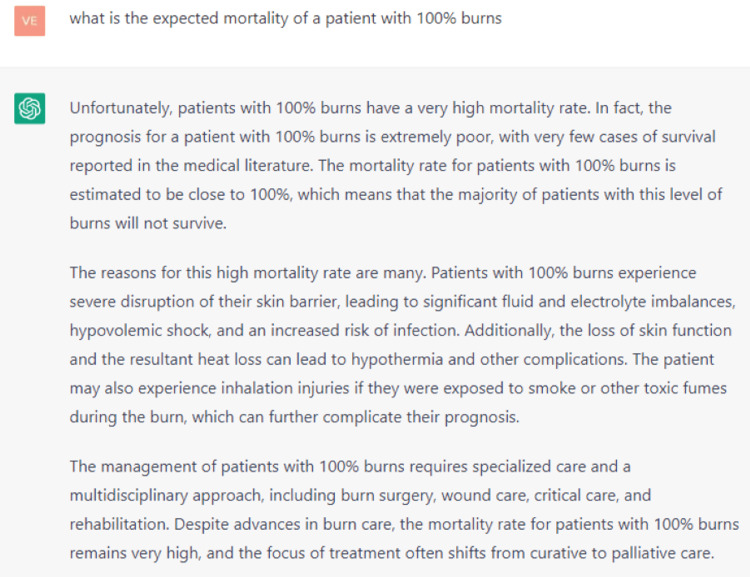
The response from ChatGPT to the query "What is the expected mortality of a patient with 100% burns?"

**Figure 3:**
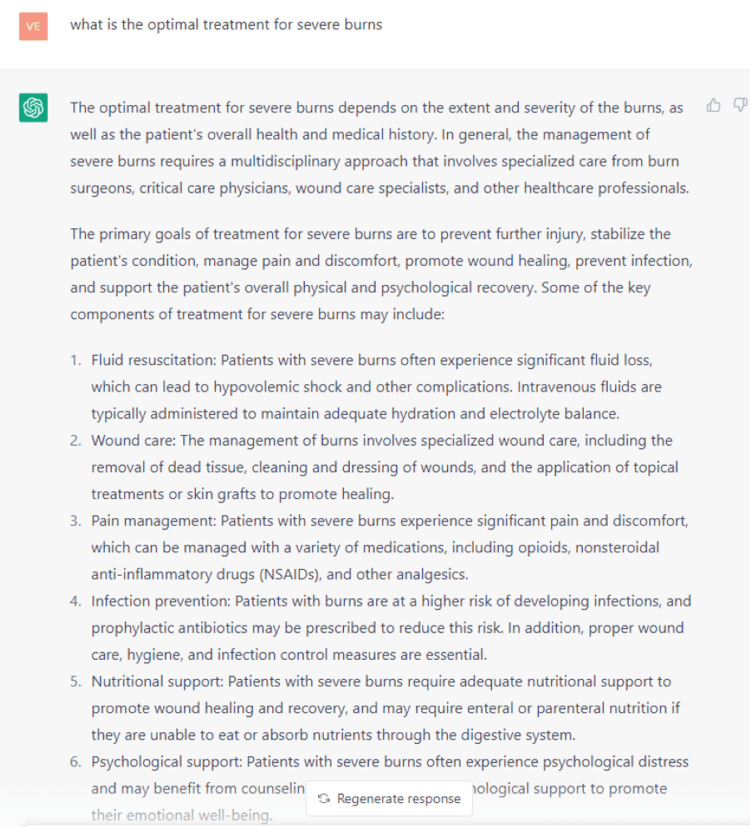
Response from ChatGPT to the query "What is the optimal treatment of severe burns?"

The requests to and responses from ChatGPT are shown in Figures 2, 3.

## Conclusions

This case report illustrates two main points. Firstly, on occasion, the capabilities of the critical care team are not sufficient to save all patients presenting to us, and when this occurs, we do not stop caring. Rather, we switch our focus from heroic measures (which, in this case, would have clearly resulted in patient suffering and a poor final result even if the patient survived) to comfort care. Secondly, the description of the illustrative case was aided by the use of ChatGPT, which was able to provide some background to the case report. From the figures we presented, it is clear that the output from ChatGPT depends very much on the questions put to it. It can provide background information, but cannot produce a proper scientific report, even when it comes to the description of a simple case. The bot provides facts, but neither synthesis nor any comment on the emotional and moral aspects of the case.
